# Prevention of post-transplant lymphoproliferative disorder in pediatric kidney transplant recipients

**DOI:** 10.1007/s00467-024-06522-2

**Published:** 2024-10-07

**Authors:** Shirley Pollack, Moran Plonsky, Rami Tibi, Irina Libinson-Zebegret, Renata Yakobov, Israel Eisenstein, Daniella Magen

**Affiliations:** 1 Technion Faculty of Medicine, Haifa, Israel; 2 Pediatric Nephrology Institute, Ruth Children’s Hospital, Rambam Health Care Campus, Haifa, Israel

**Keywords:** Post-transplant lymphoproliferative disorder, EBV infection, Graft survival, Rituximab, Pediatric kidney transplant

## Abstract

**Background:**

Post-transplant lymphoproliferative disorder (PTLD) is a devastating complication of immunosuppressive treatment in both solid organ transplantations (SOT) and hematopoietic stem cell transplantations (HSCT). Epstein-Barr virus (EBV) infection precedes PTLD in 90% of patients. Rituximab, a monoclonal anti-CD20 antibody, depletes B-lymphocytes, which are the ultimate reservoir for EBV. Although rituximab therapy is commonly used as a preventive measure for PTLD in high-risk HSCT, it is not established in SOT.

**Methods:**

Pediatric kidney transplant recipients (PKTR) underwent routine EBV-PCR surveillance. Patients with increasing viral loads, despite immunosuppressive dose reduction, were managed with preventive rituximab therapy.

**Results:**

Between 2012 and 2023, we identified eight episodes of asymptomatic EBV-PCR-positive blood tests in seven out of 65 PKTR (11%) under our care. EBV DNAemia emerged 120–720 days post-transplantation. Five of seven patients with EBV DNAemia (71%) were EBV-seronegative prior to transplantation. All five patients did not respond to MMF dose reduction and were therefore treated with preventive rituximab therapy. Following this treatment, EBV PCR clearance was observed in all patients with only minimal complications.

**Conclusions:**

PKTR who are EBV-naïve prior to transplantation are expected to have a higher prevalence of EBV DNAemia. We found that PKTR who were EBV seronegative prior to transplantation were less likely to achieve EBV clearance in response to immunosuppression dose reduction. We suggest that rituximab therapy in PKTR may be safe and effective in EBV clearance and PTLD prevention.

**Graphical abstract:**

**Figure:**
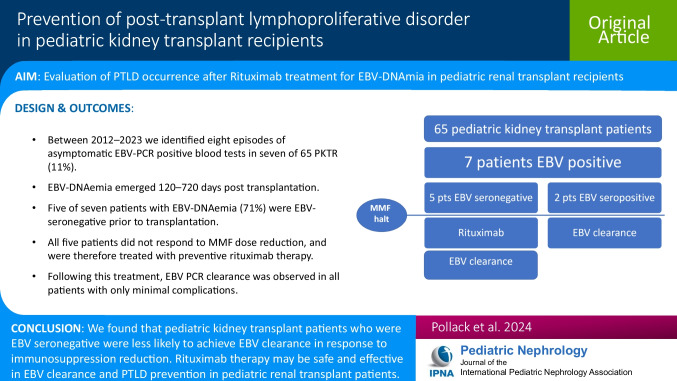
A higher resolution version of the Graphical abstract is available as Supplementary information

**Supplementary Information:**

The online version contains supplementary material available at 10.1007/s00467-024-06522-2.

## Introduction

Kidney transplantation is the preferred mode of kidney replacement therapy for kidney failure. Although lifelong immunosuppression therapy is mandatory to prevent allograft rejection and prolong graft survival, it is commonly associated with infectious complications.

Epstein-Barr virus (EBV) infection is usually transmitted via the oropharyngeal route, resulting in a polyclonal expansion of virus-containing B-lymphocytes. In immunocompetent hosts, EBV infection typically manifests as a mild, self-limited disease resembling infectious mononucleosis and is resolved through rapid elimination of EBV-infected B-lymphocytes by cytotoxic T-cells. However, in immunocompromised hosts, impaired cytotoxic T-cell function may fail to prevent massive expansion of EBV-infected B-lymphocytes, potentially leading to serious systemic EBV infection. This condition carries the risk of uncontrolled monoclonal B-cell proliferation and the development of post-transplant lymphoproliferative disorder (PTLD). PTLD is the most common malignancy in pediatric kidney transplant recipients (PKTR) [1, 2], with a reported 29-fold lifetime risk of developing PTLD in PKTR, and an eightfold risk in adult kidney transplant recipients, compared to the general population [3].

The most important risk factor for EBV infection in kidney transplant recipients is young age. While 90–95% of adults are EBV-seropositive due to past infection, younger pediatric patients are more commonly EBV-immune-naïve at the time of transplantation. Consequently, EBV-seronegative young children are particularly vulnerable to primary EBV infection and are more prone to developing PTLD. Indeed, the highest incidence of PTLD occurs during primary EBV infection rather than as a reactivation of latent EBV. This primary infection may be acquired de novo or through the transmission of infected graft lymphocyte [1].

Another risk factor for EBV infection is active CMV infection [4]. McDonald et al. [5] have shown that the prevalence of PTLD was 4.7 times higher in EBV-seronegative pediatric kidney transplant recipients compared to their EBV-seropositive counterparts. Nevertheless, there is insufficient data to predict the risk of PTLD in correlation with initial viral load, peak viral load, or the rate of viral load increase.

Since PTLD is a condition of uncontrolled proliferation of B lymphoid cells resulting from impaired T-cell function, patients under intensified immune suppression are at increased risk for PTLD. Hence, the disease is more prevalent during the first 3–12 months post-transplantation and after treatment of acute rejection episodes [6–8]. In PKTR, PTLD most commonly occurs within the first 2 years after transplantation, which aligns with the period of most intensive T-cell suppression to prevent graft rejection. Although less commonly, late-onset PTLD may occur due to lifelong immunosuppression, or after treatment of rejection episodes, accounting for fewer than 10% of cases [1, 9].

Although EBV serology is routinely utilized in the immunocompetent host to assess viral status, its use and interpretation in the immunocompromised host may be misleading due to the risk of an aborted immune response. Consequently, polymerase chain reaction (PCR)-based methods to directly quantify EBV-DNA are routinely utilized to monitor viral loads during the post-transplantation period, particularly in high-risk PKTR. Currently, there is no standardized assay for EBV quantitation [10]. The KDIGO clinical practice guideline of the care of kidney transplant recipients [11] suggests surveillance of EBV-PCR once a week for EBV-seronegative recipients during the first week post-transplantation, once monthly for the next 3–6 months, and quarterly thereafter.

Clinical manifestations of PTLD can vary from an infection-like presentation to frank lymphoma. It can progress from EBV reactivation/infection to a polyclonal disorder or to a more aggressive monoclonal disease [1].

Treatment of EBV infections primarily involves reduction of immunosuppressive therapy [11, 12]. However, while the risks of morbidity and mortality associated with PTLD are not inherently high, reducing immunosuppression entails a significant risk of graft rejection. Despite anecdotal suggestions regarding antiviral therapy for EBV infection in immune-compromised hosts [13], this approach was endorsed by the IPTA Nashville consensus conference on post-transplant lymphoproliferative disorders in pediatric solid organ transplantations (SOT) [12]. Although most patients respond to immunosuppressive dose reduction, some develop persistent EBV-DNAemia. Unfortunately, EBV-DNAemia during the initial 6 months following kidney transplantation has been identified as a risk for early graft loss [14]. Furthermore, in PKTR, chronic EBV-DNAemia during the first year post-transplantation was demonstrated to increase the risk of PTLD [14–16].

In hematopoietic stem cell transplantations (HSCT) associated with EBV-DNAemia, administration of rituximab, an anti-CD20 monoclonal antibody, targeting B-lymphocytes, has been demonstrated to shorten the duration of DNAemia and decrease the risk of overt PTLD [17]. Similarly, rituximab therapy has also shown effectiveness in adult patients with PTLD following SOT [18–21]. However, the efficacy of rituximab in the prevention of PTLD in adult or pediatric SOT with persistent EBV-DNAemia unresponsive to immunosuppression dose reduction has not been definitely established [22].

Here, we present our single-center retrospective case series detailing the outcomes of EBV infection, the development of PTLD, and allograft function in PKTR with EBV-DNAemia who were managed with preventive rituximab therapy.

## Methods

In this retrospective study, we report the protocol and outcome of preventive treatment for EBV-DNAemia in pediatric renal transplant patients under our care. Between 2012 and 2023, a total of 65 pediatric patients aged 2–19 years underwent kidney transplantation and were subsequently followed at our center. All patients received induction therapy comprising basiliximab and intravenous high-dose steroids. Maintenance immunosuppression consisted of oral steroids, with dosage tapered over the first 6 months to 5 mg/m^2^ every other day. Mycophenolate mofetil was administered at an initial dose of 600 mg/m^2^ twice daily for the first 2 weeks post-transplantation, followed by a reduction to 300 mg/m^2^ twice daily. Tacrolimus dose was adjusted to achieve target trough blood levels of 10–15 ng/ml during the first month, 8–12 ng/ml during the subsequent two months, 5–10 ng/ml during the following four months, and 5–8 ng/ml thereafter.

Anti-viral prophylaxis included valganciclovir for 3 months for patients who were seropositive for CMV prior to transplantation and for 6 months for seronegative patients.

EBV-PCR surveillance was performed bi-monthly during the first 6 months post-transplantation, monthly for the first year post-transplantation, and quarterly thereafter. PCR was performed by extracting nucleic acid (NA) from 200 µl of EDTA-uncoagulated blood samples, using either the MagNA Pure LC Total Nucleic Acid Isolation Kit with the MagNA LC2.0 instrument (Roche) (between 2012 and 8.2022), or the STARMAG 96*4 UNIVERSAL plus Kit with the Seegene STARlet IVD instrument (8.2022–2023) (Seegene). For each PCR reaction, 10 µl of the NA sample was used as a template in a 25 µl real-time PCR reaction, performed on the Rotor gene Q instrument (Qiagen) [23].

Upon detection of positive EBV-PCR results exceeding 1000 copies/μl, confirmed twice, the initial management strategy involved a 50% reduction in the MMF dose and a thorough evaluation for symptoms such as fever, abdominal discomfort, diarrhea, vomiting, anorexia, headache, and fatigue. Laboratory evaluations included complete blood count, liver function tests, kidney function tests, and inflammatory markers (CRP or ESR). EBV-PCR was repeated weekly. An increase in EBV-PCR levels during the next 4 weeks led to the cessation of MMF. These patients continued on tacrolimus (with target levels adjusted according to post-transplantation time) and prednisone (with dosages adjusted similarly). After receiving informed consent from their legal guardians, all patients exhibiting further increases in EBV-PCR levels were treated with rituximab, administered in four weekly doses of 375 mg/m^2^.

## Results

During the study period, seven out of our 65 PKTR (10.7%) experienced eight episodes of positive EBV-PCR blood tests. The patients’ ages ranged from 2 to 19 years (mean 10.5 ± 5 years). EBV-DNAemia was detected 60 to 1440 days post-transplantation (median 300 days, average 450 days). Of these seven patients, two were EBV-IgG-positive (EBNA-IgG-positive) prior to transplantation, while five were EBV-IgG-negative at the time of transplantation. All seven had received organs from deceased donors, all of whom were EBV-IgG-positive. All patients were under stable maintenance immunosuppression prior to EBV detection, and none required intensified immunosuppression prior to the onset of EBV-DNAemia. Three episodes of EBV-DNAemia were preceded by intercurrent viral infections. Whole blood EBV-PCR viral loads ranged between 3000 and 30,000 copies/ml. All patients with positive EBV-PCR were asymptomatic during the period of EBV-DNAemia. Complete blood counts revealed no cytopenias, and there was no biochemical evidence of impaired kidney function or liver function abnormalities. Cessation of MMF resulted in EBV clearance in two (25%) patients, both of whom were EBV-IgG-positive prior to transplantation. There were no episodes of graft rejection after immunosuppressive dose reduction.

Five patients (71%), who were all EBV seronegative prior to transplantation, suffered from the progressive increase of EBV load, which was unresponsive to MMF cessation. Surprisingly, these patients remained asymptomatic, with no lymphadenopathy or hepatosplenomegaly, and their blood tests were unremarkable. Imaging studies, including chest X-ray and abdominal ultrasonography, showed no evidence of PTLD. Due to persistent EBV-DNAemia, all five patients were treated with rituximab at a dose of 375 mg/m^2^/week for four consecutive weeks. All rituximab-treated patients showed EBV clearance within 7–45 days from the initiation of therapy. Adverse effects of rituximab included leukopenia in five patients (70%) and anemia in two patients (30%). There were no infectious complications and no graft dysfunction during a follow-up period of 24 to 78 months after rituximab therapy.

**Fig. 1:**
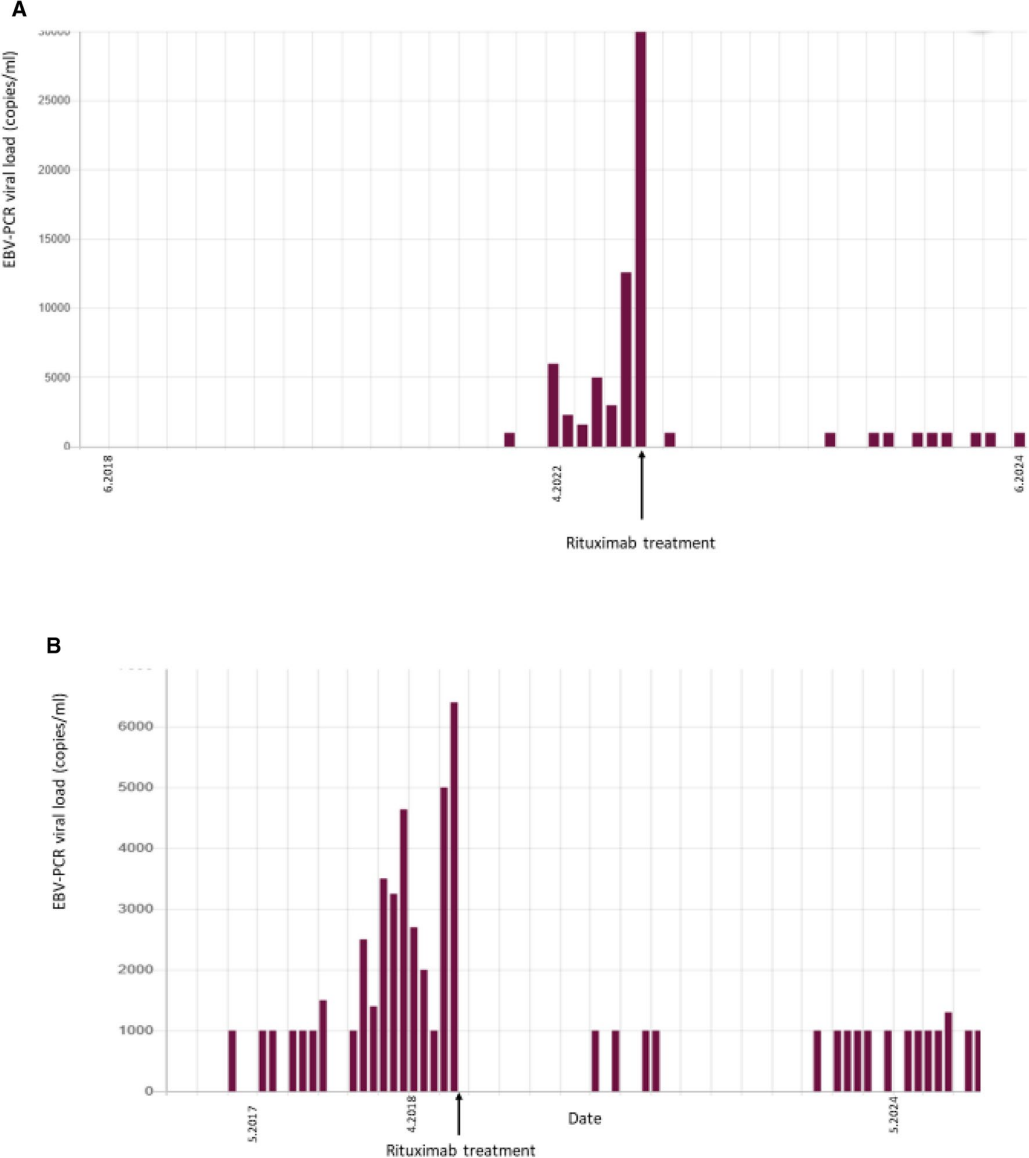
**A** EBV viral load of a pediatric kidney–transplanted patient #1 in response to rituximab therapy. **B** EBV viral load of a pediatric kidney–transplanted patient #2 in response to rituximab therapy

Figure 1 represents the timeline of EBV viral loads in response to rituximab therapy in two study participants, out of the five who received this treatment.

## Discussion

Kidney transplant recipients are predisposed to infectious complications due to their immunocompromised status. EBV-DNAemia can be particularly persistent in this population due to reduced T-cell function. EBV infection in kidney transplant recipients has the potential to progress to PTLD or premature graft loss. Nevertheless, the risk of PTLD is not well correlated with initial viral loads, peak viral loads, or the rate of viral load increase [24].

Despite the common occurrence of EBV-DNAemia in kidney transplant recipients (29–31%) [25] and even higher rates in PKTR (57%) [26], our cohort of PKTR exhibited a relatively lower prevalence at 12%. This finding is rather unexpected, particularly considering that EBV-naïve PKTR are anticipated to have a higher prevalence of post-transplantation EBV-DNAemia. Additionally, we noted that EBV-DNAemia in our cohort manifested between 120 and 720 days post-transplantation, contrasting with most reports suggesting an earlier onset within the first 3–6 months post-transplantation [25]. This disparity may be ascribed to the relatively small number of patients included in our series.

Reduction of immunosuppression is the primary recommended intervention for EBV-DNAemia in SOT [11]. In our series, consistent with the previous report by Fulchiero et al. [6], PKTR who were EBV seronegative prior to transplantation exhibited an increased risk of post-transplantation EBV-DNAemia and were less likely to achieve EBV-DNAemia clearance through this approach, thus necessitating additional treatments.

Treatment of persistent EBV-DNAemia with antiviral drugs, such as ganciclovir or valganciclovir, in combination with IVIG, has been reported in kidney transplant recipients but remains a topic of controversy [8, 14]. In HSCT recipients at high risk of PTLD, the elevated risk of graft-versus-host disease (GVHD) often complicates the reduction of immunosuppression. Consequently, rituximab has emerged as a common initial preemptive intervention in the HSCT setting [27]. However, reports on the use of rituximab in high-risk SOT adult recipients for PTLD prevention have shown increased complications and conflicting results [28, 29].

As already noted, all our PKTR treated with rituximab achieved complete clearance of EBV-DNAemia, experiencing only mild transient complications. These results are consistent with previous reports [20, 22]. Although the efficacy of rituximab therapy in preventing EBV-associated PTLD in PKTR remains to be fully elucidated, our small-scale study suggests that rituximab may be both effective and safe for this purpose. All patients in our study had undetectable EBV-DNAemia following the described treatment protocol, unlike previous reports which showed only partial resolution of EBV-DMAemia [22]. Furthermore, both Martin et al. [20] and Walti et al. [21] have also shown that rituximab, used either as induction therapy or for EBV-DNAemia prevention, was not associated with increased risk for PTLD in renal transplant recipients.

Our study has several limitations, including its retrospective design, small sample size, and the absence of a control group. Additionally, IgG levels were not monitored following rituximab treatment.

Based on our findings, we propose that rituximab therapy may be a safe and effective approach for clearing EBV in PKTR with persistent EBV-DNAemia who are unresponsive to reductions in immunosuppressive therapy. However, further evaluation through controlled, large-scale multicenter studies is warranted to validate our findings.

## Supplementary information

Below is the link to the electronic supplementary material.

Graphical abstract (PPTX 337 KB)
