# Supplementary material for: Prevention of post-transplant lymphoproliferative disorder in pediatric kidney transplant recipients
Source: Pediatr Nephrol. 2024 Oct 7;40(3):829–34. doi: 10.1007/s00467-024-06522-2 (PMC11747069; doi:10.1007/s00467-024-06522-2)
Supplement: Supplementary file 1 — Graphical abstract (PPTX 337 KB) [file 467_2024_6522_MOESM1_ESM.pptx]

## Slide 1
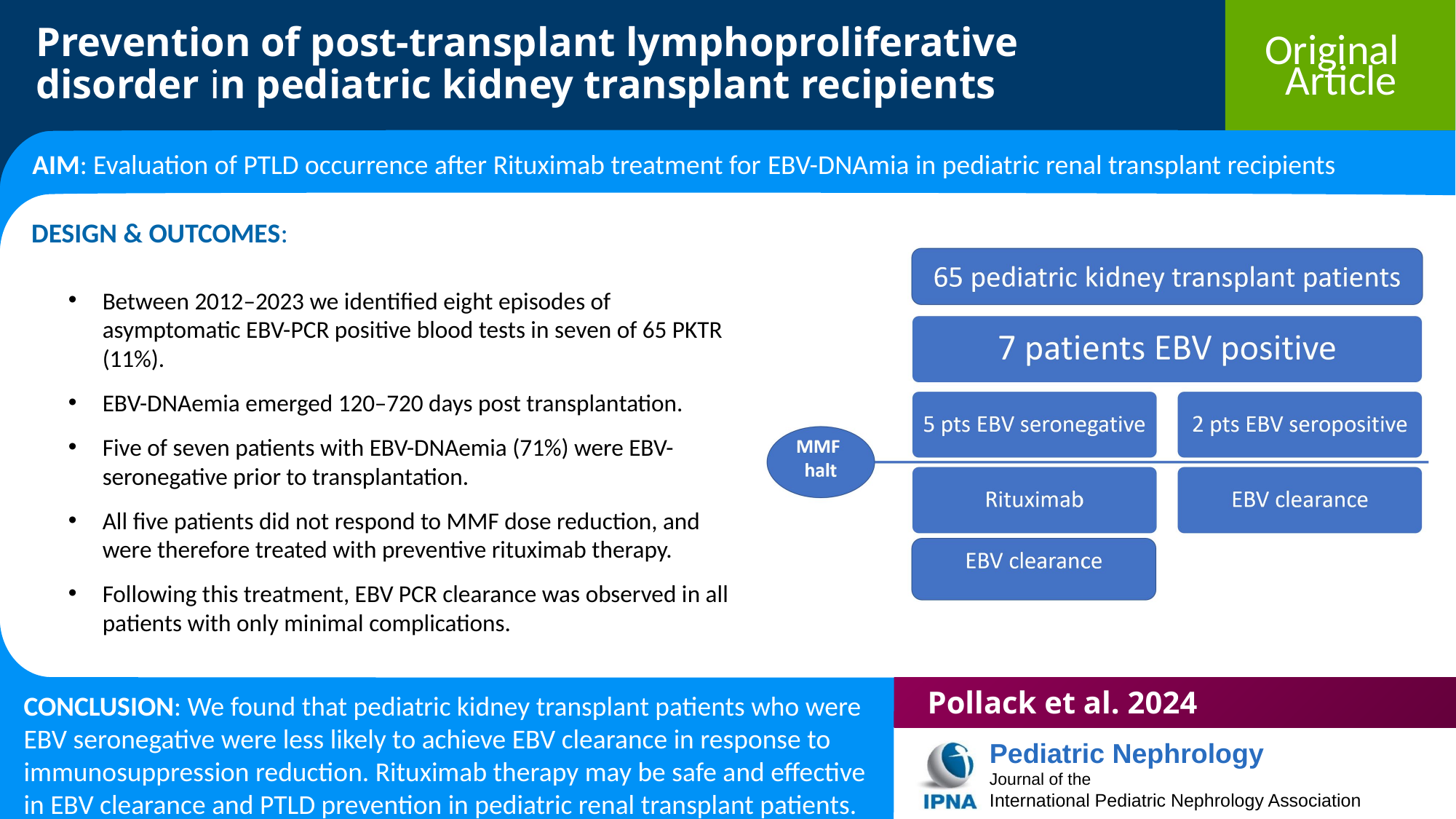

Prevention of post-transplant lymphoproliferative disorder in pediatric kidney transplant recipients
AIM: Evaluation of PTLD occurrence after Rituximab treatment for EBV-DNAmia in pediatric renal transplant recipients
DESIGN & OUTCOMES:
Between 2012–2023 we identified eight episodes of asymptomatic EBV-PCR positive blood tests in seven of 65 PKTR (11%).
EBV-DNAemia emerged 120–720 days post transplantation.
Five of seven patients with EBV-DNAemia (71%) were EBV-seronegative prior to transplantation.
All five patients did not respond to MMF dose reduction, and were therefore treated with preventive rituximab therapy.
Following this treatment, EBV PCR clearance was observed in all patients with only minimal complications.
Pollack et al. 2024
CONCLUSION: We found that pediatric kidney transplant patients who were EBV seronegative were less likely to achieve EBV clearance in response to immunosuppression reduction. Rituximab therapy may be safe and effective in EBV clearance and PTLD prevention in pediatric renal transplant patients.
